# Feasibility of Using a Cheap Colour Sensor to Detect Blends of Vegetable Oils in Avocado Oil

**DOI:** 10.3390/foods13040572

**Published:** 2024-02-14

**Authors:** Natasha D. Lorenzo, Roney A. da Rocha, Emmanouil H. Papaioannou, Yhan S. Mutz, Leticia L. G. Tessaro, Cleiton A. Nunes

**Affiliations:** 1 Department of Chemistry, Federal University of Lavras, P.O. Box 3037, Lavras 37203-202, MG, Brazil; natasha.lorenzo@estudante.ufla.br (N.D.L.); leletessaro@hotmail.com (L.L.G.T.); 2 Department of Food Science, Federal University of Lavras, P.O. Box 3037, Lavras 37203-202, MG, Brazil; roney.rocha@ufla.br (R.A.d.R.); yhan.mutz@gmail.com (Y.S.M.); 3 School of Engineering, Lancaster University, Lancaster LA1 4YW, UK; e.papaioannou@lancaster.ac.uk

**Keywords:** colour, classification, RGB, authentication, chemometrics

## Abstract

This proof-of-concept study explored the use of an RGB colour sensor to identify different blends of vegetable oils in avocado oil. The main aim of this work was to distinguish avocado oil from its blends with canola, sunflower, corn, olive, and soybean oils. The study involved RGB measurements conducted using two different light sources: UV (395 nm) and white light. Classification methods, such as Linear Discriminant Analysis (LDA) and Least Squares Support Vector Machine (LS-SVM), were employed for detecting the blends. The LS-SVM model exhibited superior classification performance under white light, with an accuracy exceeding 90%, thus demonstrating a robust prediction capability without evidence of random adjustments. A quantitative approach was followed as well, employing Multiple Linear Regression (MLR) and LS-SVM, for the quantification of each vegetable oil in the blends. The LS-SVM model consistently achieved good performance (*R*^2^ > 0.9) in all examined cases, both for internal and external validation. Additionally, under white light, LS-SVM models yielded root mean square errors (RMSE) between 1.17–3.07%, indicating a high accuracy in blend prediction. The method proved to be rapid and cost-effective, without the necessity of any sample pretreatment. These findings highlight the feasibility of a cost-effective colour sensor in identifying avocado oil blended with other oils, such as canola, sunflower, corn, olive, and soybean oils, suggesting its potential as a low-cost and efficient alternative for on-site oil analysis.

## 1. Introduction

The production of avocado oil has been steadily increasing due to its recognised health benefits, attributed to a high concentration of unsaturated fatty acids, specifically, oleic acid. Recent comparative studies have been conducted to provide qualitative and quantitative evidence of the nutritional value of avocado oil in respect to olive oil [1]. Avocado oil has been associated with the potential lower risk of chronic degenerative illnesses [2]. As a result, both avocado and olive oils are esteemed as premium delicacies due to their health benefits and are categorised as high-quality oils of considerable value. These benefits justify their popularity in the culinary world.

Due to their premium pricing, extra virgin oils are susceptible to fraudulent practices. Notably, avocado oils have been a central point of various studies investigating adulteration, blend quantification, and geographical provenance [3,4,5]. On the other hand, mixing different oils is acceptable if appropriately labelled, ensuring compliance with legislation and facilitating inspection to validate the accuracy of the label information.

Despite the analytical performance of methods commonly used to assess oil quality, especially chromatographic and spectroscopic methods [3], they are costly and necessitate trained analysts and specialised equipment. These analyses are time intensive, involve many preparatory steps and the use of chemicals, generate chemical waste, and are energy intensive [4]. Additionally, on-site analysis is often impeded due to the utilities required, and the size and the stationary nature of equipment. Therefore, the agro-food industry urges user-friendly, cost-effective, portable, non-destructive, powerful, rapid, and in-line-applicable equipment to ensure food quality and safety.

The demand for cutting-edge analytical tools has propelled the use of portable instruments of vibrational spectroscopy, including infrared spectroscopy, to the forefront of on-site quality evaluation, food authentication, and safety assurance [5,6]. The development of portable devices has facilitated the on-site analyses without requiring costly equipment. Similarly, alternative portable devices have been investigated for food analysis, such as mobile cameras [4,5,7,8,9] and optical sensors [6,10].

Optical or colourimetric sensors have been employed to assess the quality of edible oils and fats through the application of chemometric or machine-learning approaches. Sanjaya et al. (2018) used a device comprising various light-emitting diodes and a light sensor to distinguish palm oil, olive oil, sesame oil, soybean oil, and lard [11]. Furthermore, a low-cost fluorescence sensor discriminated between olive oils of different quality levels, namely, extra virgin olive oil, virgin olive oil, and lampante olive oil [12]. In another study, a hyphenated photonics sensor detected fraud in extra virgin olive oil with refined and virgin olive oils, olive-pomace olive oils, and other common edible oils [13]. Similarly, a method based on a colourimetric sensor array was used to identify extra virgin olive oil mixed with soybean oil and corn oil [14].

The advancements in technology, specifically in miniaturization and cost reduction, can be leveraged to explore alternatives with potential to be scaled up for quality assurance. In this context, a rapid and inexpensive analytical tool could be useful for assessing the quality of avocado oils, especially for detecting mixtures with cheap oils, even in a screening approach. Therefore, the main objective of this proof-of-concept study was to explore the feasibility of using a low-cost colour sensor to detect the presence of soybean, canola, corn, sunflower, and olive oils in avocado oil. This study focused on evaluating the responsiveness of the sensor output to the presence of blends in avocado oil and its potential to predict their proportion in the blend. Furthermore, the study explored the more suitable modelling approach (linear or nonlinear) and the type of illuminant (white or UV light).

## 2. Materials and Methods

### 2.1. Samples Preparation

Soybean, canola, corn, and sunflower refined oils were purchased from a local market, while virgin avocado oil and extra virgin olive oil were obtained from local producers. All oils were acquired within their expiration dates and stored at a temperature of 5 °C until subjected to analysis.

For classification purposes, the pure avocado oil was analysed in 25 replicates. Additionally, blends of avocado oil with 5%, 10%, 20%, 35%, and 50% of each oil (namely, soybean, canola, corn, sunflower, and olive oil) were analysed in five replicates, resulting in total of 125 blends (five oils × five concentrations × five replicates).

For calibration purposes, both pure avocado oil and its blends with 5% to 50% in 5% intervals of these oils were analysed in five replicates, resulting in 55 samples for each blend. The blending procedure involved weighing the respective oils in 4 mL glass vials and manually agitating them for approximately 30 s. The analyses were conducted at least 4 h after sample preparation, with no visible bubbles in the oil medium.

### 2.2. Colour Sensor Analysis

**Figure 1:**
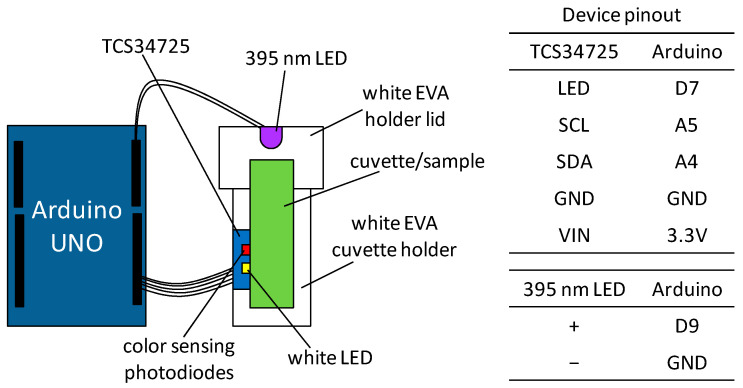
Device used to read colour parameters of oils based on a TCS34725 sensor interfaced with Arduino.

A TCS34725 colour sensor (Texas Advanced Optoelectronic Solutions Inc., Plano, TX, USA) was utilised to acquire digital readings of red, green, blue (RGB), and clear light (C) values. This sensor was interfaced with an Arduino Uno, employing an integration time of 24 milliseconds and a gain set at 1×. A sample holder was constructed using white ethylene-vinyl acetate (EVA) material. The samples, contained in 4 mL cuvettes, were analysed without the use of any solvent. The analysis of the samples took place under two distinct lighting conditions: ultraviolet light (3V, 395 nm LED) and white light from the sensor’s light source (Figure 1). The closed sample holder ensured lighting standardisation. Raw readings for red (R), green (G), blue (B), and clear light (C) outputs were acquired by averaging 10 readings. Subsequently, the raw RGB values were normalised by dividing them by the C value, resulting in the R, G, and B values used as descriptors in the models. The sensor’s output was captured using Realterm software (version 2.0.0.70, i2cchip). The Arduino code is available in the Supplementary Materials (File S1).

### 2.3. Statistical Analysis

The data analysis was explored through two main tasks: a supervised classification to discriminate blends of different vegetable oils in avocado oil and a multivariate calibration to predict the proportion of a specific oil blended with avocado oil. Two classification methods were used: Linear Discriminant Analysis (LDA) and Least Squares Support Vector Machine (LS-SVM). LDA is a technique that finds a linear combination of features that characterises or separates two or more classes, resulting in a linear classifier [15]. LS-SVM, widely used in classification and nonlinear function estimation, is a version of SVM that solves linear equations instead of a quadratic programming problem. This overcomes the major drawback of SVM, which is its higher computational burden for constrained optimisation programming [16]. In the same way, both linear and non-linear methodologies were used for the multivariate calibrations: Multiple Linear Regression (MLR) and LS-SVM. The used dataset is available in the Supplementary Materials (File S2).

#### 2.3.1. Supervised Classification

The classification task was executed using two distinct approaches: (i) a binary classification contrasting pure avocado oil against blends of avocado oil mixed with soybean, canola, corn, sunflower, and olive oils; and (ii) a simultaneous six-class classification involving pure avocado oil and each of the five oil blends. Two classification methods were employed for this task: LDA and LS-SVM, employing a radial basis function, implemented through the LS-SVM lab version 1.8 toolbox [17]. All computations were conducted using Octave version 5.2.0 [18].

The dataset was partitioned into a calibration set, encompassing 70% of the total samples, and a test set consisting of the remaining 30%, employing the Kennard-Stone algorithm [19].

The robustness of the models was assessed using the y-randomization test, which consists of fixing the X matrix (independent variables) and shuffling the y vector (dependent variables, classes) to obtain new models. It is expected that the predictive performance will decrease as the response is truly related to its predictor, thus validating the relationship between independent and dependent variables.

Model validation parameters encompassed Precision (*PRE*), Recall (*REC*), Accuracy (*ACU*), Error rate (*ERR*), F1-score (*F1S*), and Matthews Correlation Coefficient (*MCC*) [20,21] based on the total of true positives (*TP*), true negatives (*TN*), false positives (*FP*), and false negatives (*FN*):

(1) $$PRE=\frac{TP}{TP+FP}\times 100$$

(2) $$REC=\frac{TP}{TP+FN}\times 100$$

(3) $$ACU=\frac{TP+TN}{TP+TN+FP+FN}\times 100$$

(4) $$ERR=\frac{FP+FN}{TP+TN+FP+FN}\times 100$$

(5) $$F1S=\frac{2TP}{2TP+FP+FN}$$

(6) $$MCC=\frac{TP\times TN-FN\times FP}{\sqrt{\left(TP+FN\right)\left(TP+FP\right)\left(TN+FN\right)(TN+FP)}}$$

The *MCC* is a reliable statistical parameter that produces a high score only if the prediction achieves good results in all four categories of the confusion matrix (true positives, false negatives, true negatives, and false positives), proportionally to both the number of positive and negative elements in the dataset [22]. The *MCC* values can range from −1 to +1. A value of +1 indicates a perfect prediction, 0 represents a random prediction, and −1 indicates an inverse prediction.

#### 2.3.2. Multivariate Calibration

Two modelling approaches were examined to predict the proportion of soybean, canola, corn, sunflower, and olive oils blended within avocado oil: MLR and LS-SVM. All computations were conducted using Octave version 5.2.0 [18].

The dataset was partitioned into a calibration set, encompassing 70% of the total samples, and a test set, comprising the remaining 30%, employing the Kennard-Stone algorithm. The adequacy of model fitting was evaluated using the determination coefficient (*R*^2^) and the root mean squared error (RMSE) for calibration, y-randomization, and external validation (test set).

The $R_{m}^{2}$ value was computed (Equation (7)) to ensure that the predicted values obtained through external validation not only exhibit a strong correlation with the observed values but also demonstrate congruency. A threshold of 0.5 was adopted as valid [23].

(7) $$R_{m}^{2}=R^{2}\;\left(1-\sqrt{R^{2}-R_{0}^{2}}\right)$$

where $R^{2}$ and $R_{0}^{2}$ represent the quadratic correlation coefficients between the actual and predicted values, with and without the intercept, respectively.

Furthermore, the robustness of the models was examined via the y-randomization test. The $cR_{p}^{2}$ value was computed, which accounts for the distinction between the y-randomization *R*^2^ ($R_{rand}^{2}$) and calibration *R*^2^ ($R_{cal}^{2}$) (Equation (8)). A $cR_{p}^{2}$ > 0.5 was established to attest to the absence of overfitting or random adjustment [23].

(8) $$cR_{p}^{2}=R_{cal}^{2}\left(\sqrt{R_{cal}^{2}-R_{rand}^{2}}\right)$$

## 3. Results

### 3.1. Discriminating between Pure and Blended Avocado Oil

The initial approach was a binary assessment for evaluating the discriminative potential between pure avocado oil and its blends with various vegetable oils. To achieve this, blends of 5%, 10%, 20%, 35%, and 50% of these oils were denoted as the blended class, whereas pure avocado oil was considered as the pure class. The effect of the light source, whether white or UV light, on the classification outcomes was analysed. Detailed classification results are presented in Table 1 and Tables S1–S4 in the Supplementary Materials.

The differentiation between the two classes, namely pure and blended avocado oil, was attempted through LDA and LS-SVM models. The LDA models, whether employing white or UV light, exhibited unsatisfactory performance in the classification of pure avocado oil versus blended oil. The results revealed a 100% recall for the blended avocado oil class but produced a recall of 0 for pure avocado oil, both for calibration and test sets. The practical interpretation of these findings indicates that this model exhibited excessive stringency, thereby categorising all samples, including those of pure avocado oil, as blends.

Conversely, the LS-SVM model yielded praiseworthy results in the binary classification between pure and blended avocado oil (Table 1), achieving flawless classifications (with an accuracy of 100%) under white light. The *MCC* values of +1 for both calibration and test sets further corroborated these outcomes. Additionally, the low *MCC* values observed in the y-randomization test indicated the absence of overfitting or random adjustments in the model.

### 3.2. Discriminating between Blend Types in Avocado Oil

The next approach aimed to achieve multi-class discrimination between pure avocado oil and avocado oil blended with canola, sunflower, corn, olive, and soybean oils. Similar to the initial approach, discrimination among samples was attempted with LDA and LS-SVM models under two distinct illuminants (white or UV light). The performance parameters of this approach are presented in Table 2 and Tables S5–S8 in the Supplementary Materials.

The use of white light generally resulted in superior performance compared to the UV light condition across all classes, for both LDA and LS-SVM models. As in the binary classification, the LDA models exhibited inferior performance to the LS-SVM. Specifically, the LDA models had accuracies hovering around 80%; however, the low values of precision, recall, F1-score, and *MCC* indicated inadequate classification performance across all classes. Moreover, the relatively high accuracy observed in the y-randomization test suggested a potential tendency towards random adjustments. Thus, it can be inferred that the colourimetric data read by the sensor might not have been adequate to linearly describe the differentiation between pure avocado oil and its blends.

On the other hand, the LS-SVM models had superior performance, particularly under white light condition, with a low error rate across all classes. The models exhibited accuracy levels exceeding 90% for most classes in both the calibration and test sets. Furthermore, the elevated values of precision, recall, F1-score, and *MCC* confirmed the efficacy of this approach, while the poor performance in the y-randomization test, especially with low *MCC* values, suggested the absence of overfitting or random adjustments [23].

Lower *MCC* values were observed for the test set in the cases of canola (*MCC* = 0.56) and soybean (*MCC* = 0.78) blends. In these instances, a few samples assumed to be soybean blends were classified as canola oil blends (Table S2). Nonetheless, the model indicated that these samples were not pure avocado oil; rather, they lacked the specific oil blended with the avocado oil.

### 3.3. Predicting the Blend Level in Avocado Oil

In order to construct the calibration models, samples for each blend, ranging from 0% to 50% vegetable oil (canola, corn, soy, sunflower, and olive), were analysed. The statistical parameters from the MLR and LS-SVM models for all blends under the two illuminants (white and UV light) are detailed in Table 3.

The MLR models were able to predict the levels of canola, sunflower, corn, olive, and soybean oils blended with avocado oil, achieving calibration *R*^2^ > 0.8 for both illuminants (Table 3). However, the MLR models for canola and corn oil blends exhibited relatively lower calibration *R*^2^ values (0.78 and 0.66, respectively) under UV light. Also, *R*^2^ values ranging from 0.75 to 0.98 were obtained for the test set, in addition to *R*^2^*m* values > 0.5, indicating congruence between actual and predicted values. Despite high RMSE values and low *R*^2^, the *cR*^2^*p* > 0.5 suggested no overfitting or random adjustments.

**Figure 2:**
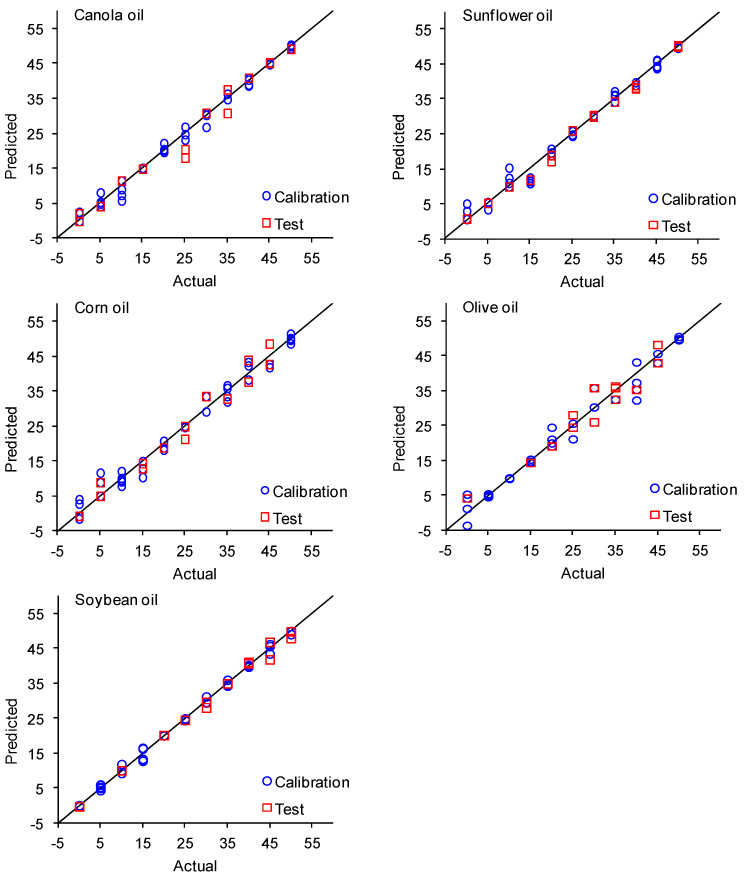
Actual and predicted proportion of canola, sunflower, corn, olive, or soybean oils blended with avocado oil using the TCS34725 colour sensor under white light and LS-SVM models.

Conversely, the LS-SVM models produced superior outcomes compared to the MLR models, particularly under white light, with *R*^2^ values > 0.93 for both calibration and test sets. The RMSE values from the LS-SVM models were notably lower than those obtained from the MLR models. Furthermore, the elevated *R*^2^*m* values (ranging from 0.87 to 0.99) indicated excellent congruence between actual and predicted values. The LS-SVM models exhibited poor performance in the y-randomization test, with *cR*^2^*p* values exceeding 0.5, suggesting the robustness of these models without overfitting or random adjustments. Comparisons between actual and predicted percentages of the vegetable oils in the blends with avocado oil by LS-SVM using white light for both calibration and test sets are presented in Figure 2.

## 4. Discussion

Certainly, the outcomes reveal that the models generated under white light conditions exhibited superior classification metrics compared to those under UV light for both calibration and test sets. A long-wavelength band at 350–420 nm in excitation and 660–700 nm in emission is attributed to the fluorescence of pigments of the chlorophyll group in olive oils [24], while excitation at 320–420 nm and emission at 400–500 nm is associated with oxidation products [25,26]. According to Hakonen and Beves [7], the fluorescence detected at a 90° angle from an excitation light might produce inner filter effects due to the presence of particles in a liquid medium. They suggested that this effect, when using a 405 nm excitation light, could provide additional informative details for distinguishing pure oils based on colour parameters obtained via a smartphone camera. However, they highlighted a possible drawback of inhomogeneous sample images, which might have contributed to the unsatisfactory classification performance for the oil blends using the colour sensor under UV light. The evident difference in performance attributed to different light sources aligns with the known significance of illumination in colourimetric techniques [26,27,28].

A noticeable disparity in classification performance and superior outcomes for LS-SVM models over LDA was evident. LDA stands out as a prevalent choice among supervised methods for tasks like food matrix authentication, characterization, and adulteration detection [29]. LDA is frequently used as a linear classifier [30], similar to MLR, a regression method relying on the linear relationship between dependent and independent variables. In contrast, LS-SVM employs a non-linear algorithm, which, although more computationally intensive, exhibits greater generalisation power [31]. Some authors emphasise that machine learning techniques such as SVM complement the implementation of low-cost sensors, compensating for limitations in their design and manufacture [32]. This is feasible because SVM operates as an optimisation-based model, seeking the best hyperplane to maximise margins within a high-dimensional space. Unlike in linear regression, where the impact of all data points is uniform, in SVM, each data point influences the final optimisation [33]. Therefore, the superiority of LS-SVM over LDA can be attributed to the mathematical qualities of each model in terms of data processing and their ability to discriminate between classes, with LS-SVM classification performing better than LDA.

These outcomes confirm that the RGB sensor coupled with LS-SVM not only detected blends of vegetable oils in avocado oil but was also able to predict the blend proportion. Assessing oil authenticity typically involves quantifying triacylglycerol components using gas chromatography or high-performance liquid chromatography, especially when evaluating blends of virgin oils, such as avocado or olive, with refined vegetable oils [34]. Another common approach involves spectroscopic methods coupled with chemometric tools [35]. However, both of these methods require the use of expensive equipment, reagents, and/or considerable time, in contrast to the colour sensor-based device used in the present work.

This study had similar or superior performance compared to previous studies that used colourimetric sensors or traditional analytical techniques to assess oil quality. Huang et al. (2022) achieved cross-validation with accuracy of 90.7% and 81.5% for distinguishing extra virgin olive oil from its mixtures with soybean and corn oil, respectively, using a colourimetric sensor array [17]. Naik et al. (2023) demonstrated that a paper-based colour sensor was able to detect the presence of palm oil and sunflower oil added to cow ghee at a concentration of 2.5% or more [36]. Amit et al. (2020) achieved an RMSE lower than 1% for a test set when using Fourier transform infrared spectroscopy to detect fried coconut oil mixed with pure coconut oil [37]. Tian et al. (2019) reported that gas chromatography can reliably identify peanut oil mixed with a minimum of 5% rapeseed oil based on fatty acid profiles [38]. Similar to the present study and others [39,40,41,42,43], all of these studies focused on demonstrating the feasibility of analytical techniques without examine mixtures of oils from different suppliers. Therefore, once the feasibility of an analytical technique has been demonstrated, further research can be conducted to validate and investigate the robustness of methods using larger and more diverse sample sets from different sources and suppliers.

## 5. Conclusions

This proof-of-concept study presents evidence regarding a cost-effective colour sensor (RGB), which can be a feasible solution for detecting and distinguishing blends of vegetable oils in avocado oil. This RGB sensor was able to quantify blends in avocado oil and their proportions.

The classification models effectively identified mixtures of various vegetable oils in avocado oil, with an accuracy exceeding 90%. Notably, non-linear LS-SVM models performed better under white light conditions, using the sensor’s light source, thereby streamlining the analysis process. In addition, the method proved to be rapid and cost-effective, without the necessity of any additional sample pretreatment.

This evidence can be used in future research for further validation and robustness studies with even higher number of samples and other oils or mixtures, thus further establishing this portable device as useful for accurately detecting oil blends.

## Figures and Tables

**Table 1:** Performance parameters for LDA and LS-SVM two-class models to discriminate pure avocado oil from its blends with canola, sunflower, corn, olive, and soybean oils using the TCS34725 colour sensor under white and UV light.

		White Light						UV Light					
		LDA			LS-SVM			LDA			LS-SVM		
		Overall	Pure	Blended *	Overall	Pure	Blended *	Overall	Pure	Blended *	Overall	Pure	Blended *
calibration	*PRE*	42.0	0.0	84.0	100.0	100.0	100.0	92.9	100.0	85.9	95.3	92.9	97.7
	*REC*	50.0	0.0	100.0	100.0	100.0	100.0	53.3	6.7	100.0	92.7	86.7	98.8
	*ACU*	84.0	84.0	84.0	100.0	100.0	100.0	86.0	86.0	86.0	97.0	97.0	97.0
	*ERR*	16.0	16.0	16.0	0.0	0.0	0.0	14.0	14.0	14.0	3.0	3.0	3.0
	*F1S*	0.46	0.00	0.91	1.00	1.00	1.00	0.52	0.13	0.92	0.94	0.90	0.98
	*MCC*	0.00	0.00	0.00	1.00	1.00	1.00	0.24	0.24	0.24	0.88	0.88	0.88
y-rand.	*PRE*	42.0	0.0	84.0	51.5	18.2	84.9	52.5	20.0	85.1	59.0	30.0	88.1
	*REC*	50.0	0.0	100.0	53.0	6.3	99.8	50.2	1.3	99.1	60.3	20.7	100.0
	*ACU*	84.0	84.0	84.0	84.8	84.8	84.8	84.4	84.4	84.4	88.1	88.1	88.1
	*ERR*	16.0	16.0	16.0	15.2	15.2	15.2	15.6	15.6	15.6	11.9	11.9	11.9
	*F1S*	0.46	0.00	0.91	0.50	0.08	0.92	0.47	0.03	0.92	0.57	0.21	0.94
	*MCC*	0.00	0.00	0.00	0.09	0.09	0.09	0.01	0.01	0.01	0.22	0.22	0.22
test	*PRE*	41.0	0.0	82.0	100.0	100.0	100.0	40.0	0.0	80.0	82.7	75.0	90.5
	*REC*	50.0	0.0	100.0	100.0	100.0	100.0	50.0	0.0	100.0	77.5	60.0	95.0
	*ACU*	82.0	82.0	82.0	100.0	100.0	100.0	80.0	80.0	80.0	88.0	88.0	88.0
	*ERR*	18.0	18.0	18.0	0.0	0.0	0.0	20.0	20.0	20.0	12.0	12.0	12.0
	*F1S*	0.45	0.00	0.90	1.00	1.00	1.00	0.44	0.00	0.89	0.80	0.67	0.93
	*MCC*	0.00	0.00	0.00	1.00	1.00	1.00	0.00	0.00	0.00	0.60	0.60	0.60

*PRE*: Precision. *REC*: Recall. *ACU*: Accuracy. *ERR*: Error rate. *F1S*: F1-score. *MCC*: Matthews correlation coefficient. * Avocado oil blended with 5, 10, 20, 35, or 50% canola, sunflower, corn, olive, or soybean oils.

**Table 2:** Performance parameters for LDA and LS-SVM six-class models to classify pure avocado oil and avocado oil blended with canola, sunflower, corn, olive, and soybean oils using the TCS34725 colour sensor under white and UV light.

		White Light													
		LDA							LS−SVM						
		Overall	Pure	Canola	Sunflower	Corn	Olive	Soybean	Overall	Pure	Canola	Sunflower	Corn	Olive	Soybean
calibration	*PRE*	62.1	82.4	42.2	100.0	68.2	80.0	0.0	98.5	100.0	90.9	100.0	100.0	100.0	100.0
	*REC*	56.8	87.5	95.0	6.7	88.2	63.2	0.0	97.8	100.0	100.0	100.0	100.0	94.7	92.3
	*ACU*	87.0	95.0	73.0	86.0	91.0	90.0	87.0	99.3	100.0	98.0	100.0	100.0	99.0	99.0
	*ERR*	13.0	5.0	27.0	14.0	9.0	10.0	13.0	0.7	0.0	2.0	0.0	0.0	1.0	1.0
	*F1S*	0.51	0.85	0.58	0.13	0.77	0.71	0.00	0.98	1.00	0.95	1.00	1.00	0.97	0.96
	*MCC*	0.49	0.82	0.50	0.24	0.72	0.65	0.00	0.98	1.00	0.94	1.00	1.00	0.97	0.96
y-rand.	*PRE*	19.2	11.8	20.7	50.0	17.7	15.3	0.0	38.2	28.2	38.7	35.2	45.3	45.2	36.8
	*REC*	16.2	12.5	46.5	3.3	22.9	12.1	0.0	36.7	76.9	75.5	8.0	15.3	18.9	25.4
	*ACU*	72.7	71.0	53.6	85.0	68.8	70.6	87.0	79.4	63.7	69.7	86.0	84.5	83.3	89.3
	*ERR*	27.3	29.0	46.4	15.0	31.2	29.4	13.0	20.6	36.3	30.3	14.0	15.5	16.7	10.7
	*F1S*	0.1	0.1	0.3	0.1	0.2	0.1	0.0	0.30	0.41	0.51	0.12	0.22	0.24	0.29
	*MCC*	0.01	−0.05	0.02	0.10	0.01	−0.04	0.00	0.26	0.28	0.36	0.15	0.22	0.23	0.28
test	*PRE*	26.6	63.6	16.7	0.0	54.5	25.5	0.0	89.7	100.0	38.5	100.0	100.0	100.0	100.0
	*REC*	41.6	77.8	80.0	0.0	75.0	16.7	0.0	86.7	100.0	100.0	70.0	100.0	83.3	66.7
	*ACU*	78.7	88.0	58.0	80.0	86.0	84.0	76.0	94.7	100.0	84.0	94.0	100.0	98.0	92.0
	*ERR*	21.3	12.0	42.0	20.0	14.0	16.0	24.0	5.3	0.0	16.4	6.0	0.0	2.0	8.0
	*F1S*	0.30	0.70	0.28	0.00	0.63	0.20	0.00	0.85	1.00	0.56	0.82	1.00	0.91	0.80
	*MCC*	0.25	0.63	0.21	0.00	0.56	0.12	0.00	0.84	1.00	0.56	0.81	1.00	0.90	0.78
		**UV Light**													
		**LDA**							**LS−SVM**						
		**Overall**	**Pure**	**Canola**	**Sunflower**	**Corn**	**Olive**	**Soybean**	**Overall**	**Pure**	**Canola**	**Sunflower**	**Corn**	**Olive**	**Soybean**
calibration	*PRE*	36.0	46.7	40.0	50.0	33.3	46.2	0.0	99.0	93.8	100.0	100.0	100.0	100.0	100.0
	*REC*	42.5	93.3	31.6	36.8	33.3	60.0	0.0	99.1	100.0	100.0	94.7	100.0	100.0	100.0
	*ACU*	81.3	83.0	78.0	81.0	80.0	78.0	88.0	99.7	99.0	100.0	99.0	100.0	100.0	100.0
	*ERR*	18.7	17.0	22.0	19.0	20.0	22.0	12.0	0.3	1.0	0.0	1.0	0.0	0.0	0.0
	*F1S*	0.38	0.62	0.35	0.42	0.33	0.52	0.00	0.99	0.97	1.00	0.97	1.00	1.00	1.00
	*MCC*	0.29	0.58	0.22	0.32	0.22	0.39	0.00	0.99	0.96	1.00	0.97	1.00	1.00	1.00
y-rand.	*PRE*	14.0	14.0	16.0	22.9	11.3	19.6	0.0	41.7	31.6	39.7	45.6	43.9	54.3	35.0
	*REC*	15.7	28.0	12.6	16.8	11.3	25.5	0.0	44.0	86.0	65.3	33.7	13.3	41.0	25.0
	*ACU*	72.2	63.4	70.8	73.4	73.4	64.2	88.0	81.7	67.6	77.1	83.3	86.1	86.0	90.3
	*ERR*	27.8	36.6	29.2	26.6	26.6	35.8	12.0	18.3	32.4	22.9	16.7	13.9	14.0	9.7
	*F1S*	0.14	0.19	0.14	0.19	0.11	0.22	0.00	0.36	0.46	0.48	0.33	0.19	0.43	0.26
	*MCC*	−0.01	−0.02	−0.03	0.04	−0.04	−0.01	0.00	0.32	0.37	0.39	0.31	0.20	0.42	0.26
test	*PRE*	34.9	45.0	14.3	28.6	100.0	21.4	0.0	52.1	63.6	25.0	62.5	66.7	33.3	61.5
	*REC*	36.7	90.0	16.7	33.3	20.0	60.0	0.0	51.4	70.0	33.3	83.3	40.0	20.0	61.5
	*ACU*	78.0	76.0	78.0	82.0	84.0	74.0	74.0	85.0	86.0	80.0	92.0	84.0	88.0	80.0
	*ERR*	22.0	24.0	22.0	18.0	16.0	26.0	26.0	15.0	14.0	20.0	8.0	16.0	12.0	20.0
	*F1S*	0.29	0.60	0.15	0.31	0.33	0.32	0.00	0.51	0.67	0.29	0.71	0.50	0.25	0.62
	*MCC*	0.23	0.51	0.03	0.21	0.41	0.24	0.00	0.42	0.58	0.17	0.68	0.43	0.20	0.48

*PRE*: Precision. *REC*: Recall. *ACU*: Accuracy. *ERR*: Error rate. *F1S*: F1-score. *MCC*: Matthews correlation coefficient. Canola, sunflower, corn, olive, and soybean oil percentages in avocado oil were 5, 10, 20, 35, or 50%.

**Table 3:** Performance parameters for MLR and LS-SVM models to predict the proportion of canola, sunflower, corn, olive, or soybean oils blended with avocado oil using the TCS34725 colour sensor under white and UV light.

		White Light		UV Light	
		MLR	LS-SVM	MLR	LS-SVM
		canola oil			
calibration	RMSE	6.02	1.44	7.60	5.37
	*R* ^2^	0.86	0.99	0.78	0.90
y-randomization	RMSE	20.97	9.48	21.40	13.52
	*R* ^2^	0.02	0.64	0.01	0.44
	*cR* ^2^ *p*	0.85	0.59	0.77	0.64
test	RMSE	6.36	2.55	7.12	7.35
	*R* ^2^	0.83	0.97	0.77	0.77
	*R* ^2^ *m*	0.70	0.97	0.57	0.60
		sunflower oil			
calibration	RMSE	3.08	1.89	2.89	2.91
	*R* ^2^	0.96	0.99	0.97	0.97
y-randomization	RMSE	22.38	6.77	21.19	10.65
	*R* ^2^	0.06	0.78	0.04	0.61
	*cR* ^2^ *p*	0.93	0.45	0.95	0.59
test	RMSE	2.63	1.37	3.86	3.81
	*R* ^2^	0.97	0.99	0.94	0.94
	*R* ^2^ *m*	0.96	0.98	0.92	0.92
		corn oil			
calibration	RMSE	4.38	2.29	8.95	4.61
	*R* ^2^	0.93	0.98	0.66	0.91
y-randomization	RMSE	23.37	6.03	19.95	8.25
	*R* ^2^	0.01	0.72	0.03	0.81
	*cR* ^2^ *p*	0.93	0.51	0.64	0.30
test	RMSE	4.31	2.62	7.53	7.91
	*R* ^2^	0.91	0.97	0.76	0.77
	*R* ^2^ *m*	0.88	0.96	0.58	0.64
		olive oil			
calibration	RMSE	7.14	2.53	6.88	4.08
	*R* ^2^	0.82	0.98	0.82	0.94
y-randomization	RMSE	22.07	11.71	21.81	13.61
	*R* ^2^	0.02	0.60	0.01	0.57
	*cR* ^2^ *p*	0.81	0.61	0.82	0.59
test	RMSE	6.39	3.07	5.74	4.92
	*R* ^2^	0.75	0.93	0.87	0.90
	*R* ^2^ *m*	0.54	0.87	0.53	0.75
		soybean oil			
calibration	RMSE	2.45	0.90	2.20	1.54
	*R* ^2^	0.98	1.00	0.98	0.99
y-randomization	RMSE	22.26	12.79	22.37	8.07
	*R* ^2^	0.03	0.40	0.01	0.63
	*cR* ^2^ *p*	0.96	0.77	0.97	0.60
test	RMSE	2.23	1.17	2.27	1.62
	*R* ^2^	0.98	0.99	0.98	0.99
	*R* ^2^ *m*	0.96	0.99	0.98	0.99

## Data Availability

Data is contained within the article or Supplementary Material.

## Supplementary Materials

The following supporting information can be downloaded at: https://www.mdpi.com/article/10.3390/foods13040572/s1, Table S1: Confusion matrix for the test set obtained by LDA two-class model to discriminate pure avocado oil from its blends with canola, sunflower, corn, olive, and soybean oils using the TCS34725 sensor under white light; Table S2: Confusion matrix for the test set obtained by LDA two-class model to discriminate pure avocado oil from its blends with canola, sunflower, corn, olive, and soybean oils using the TCS34725 sensor under UV light; Table S3: Confusion matrix for the test set obtained by LS-SVM two-class model to discriminate pure avocado oil from its blends with canola, sunflower, corn, olive, and soybean oils using the TCS34725 sensor under white light; Table S4: Confusion matrix for the test set obtained by LS-SVM two-class model to discriminate pure avocado oil from its blends with canola, sunflower, corn, olive, and soybean oils using the TCS34725 sensor under UV light; Table S5: Confusion matrix for the test set obtained by LDA six-class model to classify pure avocado oil and avocado oil blended with canola, sunflower, corn, olive, and soybean oils using the TCS34725 sensor under white light; Table S6: Confusion matrix for the test set obtained by LDA six-class model to classify pure avocado oil and avocado oil blended with canola, sunflower, corn, olive, and soybean oils using the TCS34725 sensor under UV light; Table S7: Confusion matrix for the test set obtained by LS-SVM six-class model to classify pure avocado oil and avocado oil blended with canola, sunflower, corn, olive, and soybean oils using the TCS34725 sensor under white light; Table S8: Confusion matrix for the test set obtained by LS-SVM six-class model to classify pure avocado oil and avocado oil blended with canola, sunflower, corn, olive, and soybean oils using the TCS34725 sensor under UV light; File S1: Arduino code for TCS34725 sensor; File S2: Dataset with sensor readings, oil classes and percentages.
