# Supplementary material for: Feasibility of Using a Cheap Colour Sensor to Detect Blends of Vegetable Oils in Avocado Oil
Source: Foods. 2024 Feb 14;13(4):572. doi: 10.3390/foods13040572 (PMC10888341; doi:10.3390/foods13040572)
Supplement: Supplementary file 1 [file foods-13-00572-s001.zip › supplementary_data.pdf]

Feasibility of using a cheap colour sensor to detect blends of vegetable oils in avocado oil

Natasha Lorenzo, Roney A. da Rocha, Emmanouil H. Papaioannou, Yhan Mutz, Leticia Tessaro, and Cleiton Nunes

Supplementary material

Table S1. Confusion matrix for the test set obtained by LDA two-class model to discriminate pure avocado oil from its blends with canola, sunflower, corn, olive, and soybean oils using the TCS34725 sensor under white light.

	pure	blended*
pure	0	9
blended*	0	41

*avocado oil blended with 5, 10, 20, 35, or 50 % canola, sunflower, corn, olive or soybean oils.

Table S2. Confusion matrix for the test set obtained by LDA two-class model to discriminate pure avocado oil from its blends with canola, sunflower, corn, olive, and soybean oils using the TCS34725 sensor under UV light.

	pure	blended*
pure	0	10
blended*	0	40

*avocado oil blended with 5, 10, 20, 35, or 50 % canola, sunflower, corn, olive or soybean oils.

Table S3. Confusion matrix for the test set obtained by LS-SVM two-class model to discriminate pure avocado oil from its blends with canola, sunflower, corn, olive, and soybean oils using the TCS34725 sensor under white light.

	pure	blended*
pure	9	0
blended*	0	41

*avocado oil blended with 5, 10, 20, 35, or 50 % canola, sunflower, corn, olive or soybean oils.

Table S4. Confusion matrix for the test set obtained by LS-SVM two-class model to discriminate pure avocado oil from its blends with canola, sunflower, corn, olive, and soybean oils using the TCS34725 sensor under UV light.

	pure	blended*
pure	6	4
blended*	2	38

*avocado oil blended with 5, 10, 20, 35, or 50 % canola, sunflower, corn, olive or soybean oils.

Table S5. Confusion matrix for the test set obtained by LDA six-class model to classify pure avocado oil and avocado oil blended with canola, sunflower, corn, olive, and soybean oils using the TCS34725 sensor under white light.

	pure	canola	sunflower	corn	olive	soybean
pure	7	1	0	0	1	0
canola	1	4	0	0	0	0
sunflower	3	7	0	0	0	0
corn	0	0	0	6	2	0
olive	0	0	0	5	1	0
soybean	0	12	0	0	0	0

Canola, sunflower, corn, olive, and soybean oil percentages in avocado oil were 5, 10, 20, 35, or 50 %.

Table S6. Confusion matrix for the test set obtained by LDA six-class model to classify pure avocado oil and avocado oil blended with canola, sunflower, corn, olive, and soybean oils using the TCS34725 sensor under UV light.

	pure	canola	sunflower	corn	olive	soybean
pure	9	0	0	0	1	0
canola	3	1	0	0	2	0
sunflower	0	1	2	0	3	0
corn	3	2	1	2	2	0
olive	0	1	1	0	3	0
soybean	5	2	3	0	3	0

Canola, sunflower, corn, olive, and soybean oil percentages in avocado oil were 5, 10, 20, 35, or 50 %.

Table S7. Confusion matrix for the test set obtained by LS-SVM six-class model to classify pure avocado oil and avocado oil blended with canola, sunflower, corn, olive, and soybean oils using the TCS34725 sensor under white light.

	pure	canola	sunflower	corn	olive	soybean
pure	9	0	0	0	0	0
canola	0	5	0	0	0	0
sunflower	0	3	7	0	0	0
corn	0	0	0	8	0	0
olive	0	1	0	0	5	0
soybean	0	4	0	0	0	8

Canola, sunflower, corn, olive, and soybean oil percentages in avocado oil were 5, 10, 20, 35, or 50 %.

Table S8. Confusion matrix for the test set obtained by LS-SVM six-class model to classify pure avocado oil and avocado oil blended with canola, sunflower, corn, olive, and soybean oils using the TCS34725 sensor under UV light.

	pure	canola	sunflower	corn	olive	soybean
pure	7	1	0	0	0	2
canola	1	2	2	0	0	1
sunflower	0	0	5	0	1	0
corn	1	1	1	4	1	2
olive	2	2	0	0	1	0
soybean	0	2	0	2	0	8

Canola, sunflower, corn, olive, and soybean oil percentages in avocado oil were 5, 10, 20, 35, or 50 %.
